# Evaluating the Acceptance of Hemp Food in Australian Adults Using the Theory of Planned Behavior and Structural Equation Modelling

**DOI:** 10.3390/foods10092071

**Published:** 2021-09-02

**Authors:** Debra Ann Metcalf, Karl K. K. Wiener, Anthony Saliba, Nicole Sugden

**Affiliations:** 1 School of Psychology, Charles Sturt University, Wagga Wagga, NSW 2678, Australia; kwiener@csu.edu.au (K.K.K.W.); asaliba@csu.edu.au (A.S.); nisugden@csu.edu.au (N.S.); 2 Graham Centre for Agricultural Innovation, Wagga Wagga, NSW 2678, Australia

**Keywords:** hemp food, novel food acceptance, theory of planned behavior, structural equation modeling, food choice behavior, food choice intentions, food choice, barriers and drivers to novel food acceptance

## Abstract

This research presents a mixed methods (qual-QUANT) approach to the evaluation of the intention to consume hemp foods in an Australian sample soon after its legalization, using the Theory of Planned Behavior (TPB). Structural equation modeling was used to evaluate items developed from semi-structured interviews, with a focus on the TPB factors; attitudes toward hemp food consumption, subjective beliefs, and perceptions of control. Findings support the notion that consumers may be confused about associations between Cannabidiol (CBD) oil, tetrahydrocannabinol (THC), and hemp food produced from *Cannabis sativa*. Highly salient negative associations are mediated by the perception of positive aspects of CBD for some consumers, but the value placed on others’ acceptance of hemp food is the greatest indicator of intention to consume hemp food products. It is suggested that greater education of consumers might allay fears borne of association of hemp food to either CBD or THC, and any move toward disassociation of hemp food to either entity would have positive repercussions for the hemp food industry. Findings have implications for other novel foods that carry highly salient negative associations for consumers.

## 1. Introduction

Understanding the evaluative processes in consumers’ decisions to sample a novel food has received a great deal of attention in recent times [1,2,3]. Increased use of novel technology in food production, increased globalization and exposure to new markets for consumers, and a need to develop foods with greater environmental sustainability provides a potentially increased market for novel food products. Understanding consumer perceptions and attitudes toward novel foods have become an integral component of the development process for these products to succeed in the market [4,5]. There are several standardized psychometric instruments that evaluate discrete factors of novel foods, general food choice, and personal attributes which might influence novel food acceptance but there is not currently a standardized instrument that allows evaluation of intention to consume foods that applies broadly across all types of novel foods. A common framework employed in the evaluation of discrete novel foods is the Theory of Planned Behaviour [6,7]. Consistent with other studies [8,9] the current research has utilized a two-stage approach to item development and data collection under the TPB framework to evaluate consumers’ intention to consume hemp food.

Hemp food produced from the seeds of the plant *cannabis sativa* is a recent introduction to the Australian market, having passed legislative requirements for approval as a novel food in November 2017 [10]. Hemp food was previously illegal in Australia due to a perception of issues related to controlling product levels of tetrahydrocannabinol (THC), the psychoactive property of cannabis. As a consequence, the approval of hemp food now includes a requirement that all production, importing, and manufacture of hemp food products undergo a stringent compliance process aimed at keeping THC levels well below prescribed percentages that would potentially induce a psychoactive effect in the consumer [11]. Research of the effects of THC in hemp food at designated percentages (0.001% legal limit and twice this concentration) has determined that “consumption of low-content THC oil does not result in positive biological assessments” [12] (p. 101). Hemp food is marketed to consumers as having several health benefits and promoted to producers as an environmentally sustainable and high-yielding crop. Despite the purported benefits, however, hemp food does not seem to have infiltrated the Australian market to a degree that might be expected [13]. Hemp food presents as a unique type of novel food, with many associations to other uses of cannabis from building products and textiles to medicinal products and illicit uses [14]. Evaluating the impact these associations might have on the acceptance and intention to consume hemp food has the potential to not only assist the Australian hemp food industry to better understand consumer perceptions of their products but may also provide a protocol for evaluation of novel foods to be developed in the future. As innovation in food production increases to meet the demands of sustainability and increasing populations, early evaluation of potential novel foods is essential.

### 1.1. Theoretical Background

The theory of planned behavior posits that intention to perform a behavior is correlated to actual behavior in an extension to the theory of reasoned action [6] where behavioral and normative beliefs are reported to contribute to attitudes toward a behavior, and subjective beliefs form through perceptions of societal norms regarding that behavior Cognitive evaluations of these attitudes and beliefs contribute to consumers’ intention to perform the behavior themselves. Expanding on this, TPB includes volitional control as an additional consideration, reporting that people must have a perception of control over the behavior if the intention is to be realized as actual behavior [7]. Several hundred studies have applied TPB within a variety of disciplines with accuracy [7] and the utility of the theory in the evaluation of novel food acceptance has been supported by experimental validation of the intention–behavior relationship [1,8,15]. A review of 42 studies employing TPB as a measure of intent to consume a discrete food while evaluating the intention–behavior relationship reported a strong association overall (*r* = 0.45) between intention to consume and actual consumer behavior.

### 1.2. Methodology

There is currently no standardized psychometric instrument available that is appropriate for capturing the unique associations attributed to a discrete novel food within a TPB framework. Consistent with previous novel food research this study has developed a fit-for-purpose scale for use in the study. Examination of currently available literature did not discover adequate research on consumer perceptions and attitudes toward hemp food to enable the development of the TPB items without consumer consultation. As a result, a mixed-method (qual-QUANT) approach as described by Teddlie and Tashakkori [16] was adopted for the project which was conducted under a pragmatic framework. While pragmatism is more of a philosophical framework than a methodology, it allows for the methodology appropriate to each phase to be applied and a re-evaluation of the findings from each to form meta-inferences beyond those yielded from the findings within each phase. The first phase consisted of semi-structured interviews which informed the development of TPB items, and the second phase employed the TPB items in a questionnaire that was disseminated online nationally to evaluate consumer acceptance of hemp food in the Australian market.

## 2. Materials and Methods

### 2.1. Phase 1-Interviews

Approval for the semi-structured interviews was granted by the Charles Sturt University Human Ethics Committee protocol H18201 and was conducted in adherence to the rules of the Declaration of Helsinki of 1975, and 2013 revision.

#### 2.1.1. Participants

Fourteen participants responded to a snowball request on social media and consisted of adults from across the greater Melbourne area who were willing to travel to the designated interview address. Selection criteria included being over 18 years of age and having some degree of responsibility for the food preparation and purchasing decisions in their household. Each person received a $50 Coles-Myer gift voucher for their participation, to ensure that travel costs did not discourage participation. Participant payments and hire of the interview venue were funded via a member grant awarded by the Charles Sturt University Graham Centre for Agricultural Innovation.

#### 2.1.2. Apparatus

Semi-structured interviews were conducted by the first author using a guide sheet for topics relating to hemp food, general attitudes toward food, and current food behaviors. General topics and questions were compiled in a collaborative exercise between the authors from findings of a literature review of novel food acceptance, and in consideration of the theoretical guidelines [6]. Questions to guide the interviews included:

- Prior to this study what did you know about hemp food?
- How do you feel about the legalization of hemp food?
- What advantages/disadvantages do you see in consuming hemp food?
- What are the factors you take into account before trying a new food?
- How does price impact your food decisions?
- Do you have medically directed food limitations?
- How would you decide your relationship with food?

The subject of THC in hemp food was only broached if the participant had not already introduced the topic. Participants were asked to provide any information they thought relevant to their food choice and discuss why they would or would not be likely to consume hemp food.

#### 2.1.3. Procedure

Interviews were conducted in Greensborough, Victoria, with the number of participants determined through saturation [17]. Saturation was deemed to have been reached when no new themes were identified for four consecutive participants. The number of interviews before saturation was reached in the current study (*n* = 14) is consistent with evidence that 12 are sufficient [17]. Interviews with participants lasted between 20–30 min beyond the time it took to complete participant information and consent materials. Participants were invited to speak freely on topics and were invited to elaborate further when the interviewer identified a potential new theme from the discussion. Data was transcribed using NCH Express Scribe^®^ software and manually analyzed twice using thematic analysis. The first analysis occurred at the time of completion of each interview and involved the manual generation of semantic codes from the interviews as described in [18]. Five broad codes were identified and consisted of:

- Concern over failing a roadside drug test
- Health benefits to consuming hemp food
- No knowledge that hemp food is legal
- Hemp is a by-product of marijuana
- Regard of hemp as a supplement, not a food staple

The second analysis was undertaken as a review of the transcripts after completion of all of the interviews. No additional themes were discovered in the second analysis although some themes were further elaborated through analysis of latent meanings. A critical realist approach, together with guidelines for the development of TPB items [7], was employed in the development of the items for inclusion in the next phase of the study. The process of thematic analysis was undertaken in adherence to the guidelines for good thematic analysis as described in [18] (p. 33). Where responses described a subjective, normative, or control belief the data was compiled into a list of statements for development into TPB items; this is described further in the results section. Where a response did not prescribe itself to suitability for evaluation under TPB the data was set aside for evaluation as a separate construct in the questionnaire.

### 2.2. Phase 2–Online Questionnaire

Approval for the second phase was granted by Charles Sturt University Human Ethics Committee, protocol H18273 under the rules of the Declaration of Helsinki of 1975, and 2013 revision.

#### 2.2.1. Soft Launch Data

Prior to the final dissemination of the questionnaire, analysis of an online soft launch provided 329 cases (participants were aged 18–74, *M* = 29.75, *SD* = 15.22 years, males *n* = 123, females *n* = 206) for which initial internal consistency and correlational data were obtained. Cronbach’s alpha indicated acceptable internal consistency reliability for four of the five constructs and statistics are reported in Table 1. Cronbach’s alpha for control beliefs was less than ideal, and it was reported that removal of an item “I have seen a place where hemp food is sold” would increase the alpha value to a more acceptable 0.650, however, it was determined that the item had utility as a stand-alone item even if it were to be removed from the final analysis so was retained for inclusion in the questionnaire. Examination of the correlation structure revealed almost all item correlations were significant and greater than 0.3 with a large number between 0.5 and 0.8. The items were deemed satisfactorily representative of the intended TPB constructs and full Phase II data collection proceeded.

#### 2.2.2. Participants

The final dataset included all cases from the soft launch, as explained above, as well as the remainder of the datasets collected to fulfill the predetermined quota. A total of 2224 cases remained after removal of multivariate outliers with Mahalanobis’ distances greater than 49.73, the *df* value for 23 items using a significance value *p* < 0.001. No univariate outliers with z-scores greater than 3.29 were detected in subscales for a positive attitude, negative attitude, subjective norms, or intention to consume. The control belief items “I am free to choose the food I wish to eat” and “Choosing hemp food is a choice I am free to make” reported univariate outliers for those participants who disagreed with these statements. This occurred because the majority of respondents strongly agreed with the statements, resulting in strongly skewed results. These items were retained, however, as removing them would have removed only those participants for each item who felt strongly either way about control of their food choices and unduly influenced the total score for perceived control, distorting the outcome. Inspection of probability plots for the two items indicated these items held a degree of linearity that was sufficiently normal for inclusion in SEM procedures as described in [19] (p. 103).

Participants were aged 18–74 (*M* = 42.84, *SD* = 14.57) years and consisted of 942 (42.3%) males and 1284 (57.3%) females. 843 participants (37.9%) held a bachelor degree or higher, 617 (27.7%) held a trade certificate or equivalent, 499 (22.4%) completed high school, and 267 (12%) did not complete high school.

Analysis of the data proceeded in accordance with guidelines for item development [19] resulting in the dataset being split into three equal samples. The first sample was subjected to exploratory factor analysis (EFA) to provide an initial theory-driven model, the second was subjected to confirmatory factor analysis (CFA) to evaluate the statistical performance of the model, and the third sample provided retest reliability for the model and data for analysis. The current research uses structural equation modeling (SEM) techniques in the final analysis. The final dataset was split into three approximately equal for age, gender and educational attainment smaller datasets for analysis using the SPSS split file function. Descriptions for each of the randomly allocated datasets are provided in Table 2.

#### 2.2.3. Apparatus

To provide suitable data to determine the equal samples, participants were asked questions pertaining to age, gender, postcode, and education attainment. A single question to determine if participants had known that hemp food was a legal product in Australia before hearing about the current study sought to evaluate the spread of awareness since legalization. This question was a direct result of none of the participants in Phase I have been aware that hemp food had been legalized before seeing the invitation to participate on social media. The TPB items as developed in the study and previously described for the soft launch (Table 4) were also included in the online questionnaire. Internal reliability statistics for the TPB items were deemed acceptable for each of the analyses and are reported in Table 3.

#### 2.2.4. Procedure

The questionnaire was disseminated via Qualtrics^®^ online survey platform under the same terms and conditions as for the soft launch with de-identified data downloaded for analysis using SPSS^®^ version 27 for EFA, and IBM Amos version 27 for CFA and SEM analyses.

## 3. Results

### 3.1. Phase 1-Interviews

#### 3.1.1. Thematic Analysis

Themes were identified initially in list format until no new themes were revealed by four subsequent participants. In the second round of the analysis, each theme was further evaluated to ascertain alignment with the theory of planned behavior, assigning topics to the TPB categories of behavioral beliefs, normative beliefs, and control beliefs. The determination of themes for inclusion in the survey and the exact wording of each of the items proceeded as a collaboration between the researchers. Examples of how this was applied included:

“Concern over failing a roadside drug test”.

Concern over failing a roadside drug test was a theme common amongst participants. Despite assurances given by participants that they felt comfortable at the idea of consuming hemp food, this was often contradicted later in the interview when statements were further qualified, for example where one participant stated: “provided I didn’t have to work or drive the next day”. This response was further probed with the participant revealing that he was not convinced that he would not be somehow affected by hemp food, referring to the relationship that hemp has with marijuana and the psychoactive properties as the reason. Despite this, the participant remained adamant that he would try hemp food.

The resulting item appeared in the survey as “Eating hemp food might result in a positive roadside drug test” to which participants rated their degree of agreement.

“Health benefits to consuming hemp food”.

Participants displayed a general tendency toward a belief that hemp food offered many health benefits. However, it was difficult to discern how much of this belief was the result of internet browsing by the participant before the interview. None of the participants reported knowing that hemp food had been legalized in Australia prior to the hearing of our study and was not able to specify particular health benefits. Where participants confessed to having only read of the health benefits on the internet prior to the interview their response was recorded as knowledge rather than subjective belief. Some participants, however, associated hemp food with medicinal cannabis, also known as cannabidiol (CBD) oil, and made the association of health benefits based on that. Four items were developed relating to this theme which reflected the positively perceived attributes of hemp food. “Eating hemp food is healthy; Eating hemp food would make a person feel more relaxed; Eating hemp food would likely reduce anxiety; There are many benefits to eating hemp food” Participants rated each statement on how much they agreed with them.

#### 3.1.2. Confirmation of the Items

Response items were developed as a collaborative exercise between researchers to reflect the TPB constructs of behavioral and normative beliefs and perceived control as described in [7]. Behavioral beliefs were further defined as either contributing to a positive or negative attitude. Previous studies using TPB have found positive and negative attitudes toward novel food to have differential influence on their acceptance, leading to occasion for evaluation of the oppositely polarized viewpoints as a discrete entity. For example, [2] found negative attitudes toward insect-eating behavior to be a significant barrier to insect consumption after perceived control and food neophobia. [8] (p. 32) reported perceived control to be a “weak determinant of intentions” however when disgust toward seeing insects was incorporated into the control construct, it correlated negatively with intentions, *r* (231) = −0.58, *p* < 0.001. Measuring disgust as it applies specifically toward insect-eating behavior and evaluating this construct within a negative behavioral belief paradigm might have provided clearer insight into attitudes toward eating insects.

While not using TPB as the theoretical framework per se, [20] reported that negative perception of factors related to genetically modified (GM) foods were critical to acceptance. It has previously been suggested that highly salient negative factors relating to hemp food’s association with alternate uses for cannabis have a greater influence on early acceptance and uptake of the novel food than positive health benefits [13].

Consistent with a previous approach [2], negative behavioral beliefs have been treated as a discrete construct in the current study resulting in four constructs to be evaluated against intention; negative behavioral beliefs, positive behavioral beliefs, normative beliefs, and perceived control. Response items were then evaluated by four third-party volunteers who gave their interpretation of each item including whether they considered the behavioral beliefs to be a positive or negative attribute of hemp food, and this was compared to the researchers’ transcript for confirmation. Once it was agreed that the interpretation was consistent with intended meanings, the items were transposed into the phase 2 questionnaire. Items to measure intention were developed in accordance with guidelines for measuring intention in TPB surveys [7]. The final 25 TPB response items are included in Table 4. Items were scored on a scale from 1 = strongly disagree, to 7 = strongly agree. The seven-point scale is consistent with the recommendations of the TPB theory principal [7] (p. 120) and others who have utilized TPB for the evaluation of food choice behavior [9].

### 3.2. Results Phase 2

#### 3.2.1. Item Reduction

Exploratory factor analysis was conducted to determine whether each of the constructs to be evaluated under structural equation modeling techniques met the requirements of loading onto a single factor as described in [19]. Preliminary analyses were conducted on the first data subset (EFA sample, *n* = 731) for normality and suitability of the data for factor analysis. Each subscale was subjected to maximum likelihood extraction with direct oblimin rotation to confirm the existence of a unidimensional factor and suitability for SEM analysis. This resulted in the removal of the item “*I have seen a place where hemp food is sold*” from the control beliefs construct as it did not load on the main factor. This item might have been viewed by consumers as less within their control than the other control items and separate from their volition over hemp consumption.

Two items from the positive attitude construct (PA3: *Eating hemp food would make a person feel more relaxed* & PA4: *Eating hemp food would likely reduce anxiety*) also did not load on the main factor. Two discrete factors were confirmed through the calculation of eigenvalue cut-offs using the Monte Carlo calculator for five items, 731 response sets, and 100 iterations. The two items forming the second factor were, however, theoretically important to the final analysis and reported correlations with the remaining items between 0.3 and 0.5. Therefore, these items were retained and defined as a separate construct titled CBD in reference to the consumer perceptions of Cannabidiol and hemp oils being the same or similar entities. The items reflected perceptions of the psychological benefits of consuming hemp food that resulted from the association of CBD oil and hemp oil. While a minimum of three items is preferred for analysis using SEM techniques, it is reported that two items are acceptable “when the variables are highly correlated with each other (r > 0.70)” [21] (p. 80) and this criterion was satisfied (*r* = 0.75, *p* < 0.001).

#### 3.2.2. Item Confirmation

An a priori model was constructed from the findings of EFA and evaluated using CFA on the second data subset (CFA sample, *n* = 727). Model output revealed a significant Chi-square fit statistic (741.505, *p* < 0.001). While significant Chi-square goodness of fit values can be an indication of a poorly fitting model, it has been found to be sensitive to large sample sizes [22] as is the case in the current study. The model reported a marginally acceptable RMSEA (0.076, CI = 0.071–0.082), and similarly marginal model fit indices (NFI = 0.926, TLI = 0.927, CFI = 0.939). Examination of the modification indices demonstrated an unexpected relationship between the CBD construct, negative attitude, and positive attitude where CBD was reported to co-vary positively with each of the attitude constructs, yet they reported a negative correlation between themselves. Similarly, there was a positive correlation between CBD and each of the attitude constructs, and positive and negative attitudes reported a negative correlation. Values for these are demonstrated in Table 5.

A positive association between CBD and positive attitudes was expected, as was the negative relationship between positive and negative attitudes (Table 5). However, the positive relationship between CBD and negative attitude constructs was not expected given that the CBD construct was comprised of items originally included in the positive attitude construct and that loaded onto the same factor as the remaining positive attitude items. It was determined, therefore, that CBD might act as a mediator between negative attitude and intention rather than have a direct influence. The items comprising the CBD construct were removed from CFA and set aside for inclusion in the final model as a mediating construct, and the model rerun.

The CFA model was further adjusted after examination of the error terms of items SN1 (My family would think it is okay for me to consume hemp food), SN2 (My friends would think it is okay for me to consume hemp food), and SN3 (My peers would approve if I consumed hemp food) being covaried. Similarly, items Con1 (I am free to choose the food I wish to eat), and Con2 (Choosing to eat hemp food is a choice I am free to make) were also covaried. These modifications were theoretically supported and the structure was carried forward to the subsequent modeling phase.

**Figure 1:**
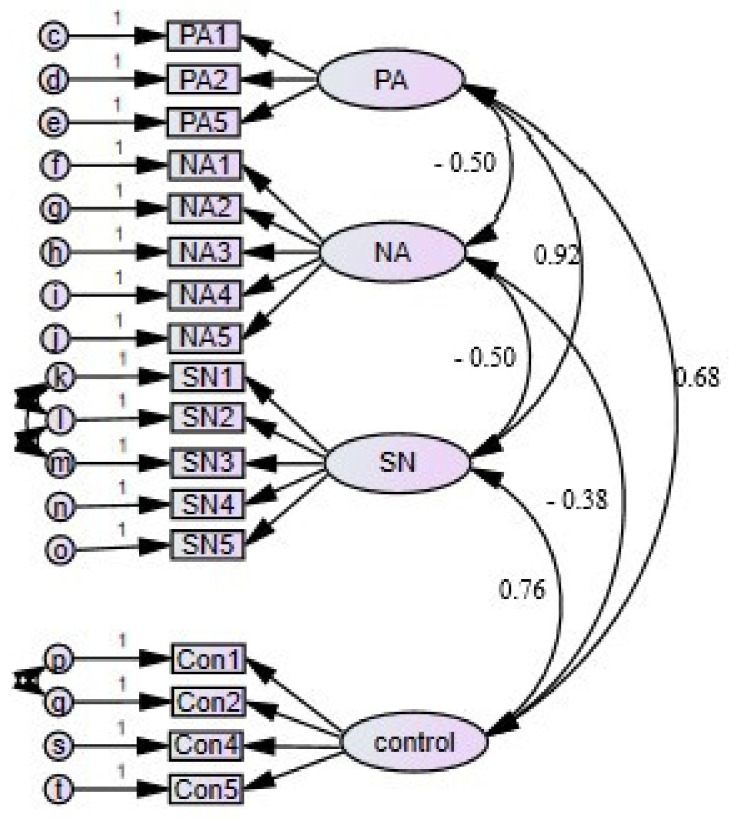
CFA of TPB items with standardized values for constructs.

Model fit for the final CFA model was deemed acceptable for analysis using structural equation modeling with the CBD construct to be inserted into the model as a mediator. The Chi-square fit statistic remained significant (395.310, *p* < 0.001), however its sensitivity to large sample sizes was considered the reason for the significance as all other appropriate fit indices [23] indicated the model was a good fit to the data, RMSEA = 0.060, (CI = 0.054–0.067), NFI = 0.956, TLI = 0.959, CFI = 0.967. The final CFA model is pictured in Figure 1.

#### 3.2.3. Modelling Intention to Consume Hemp Food Using the Theory of Planned Behavior

**Figure 2:**
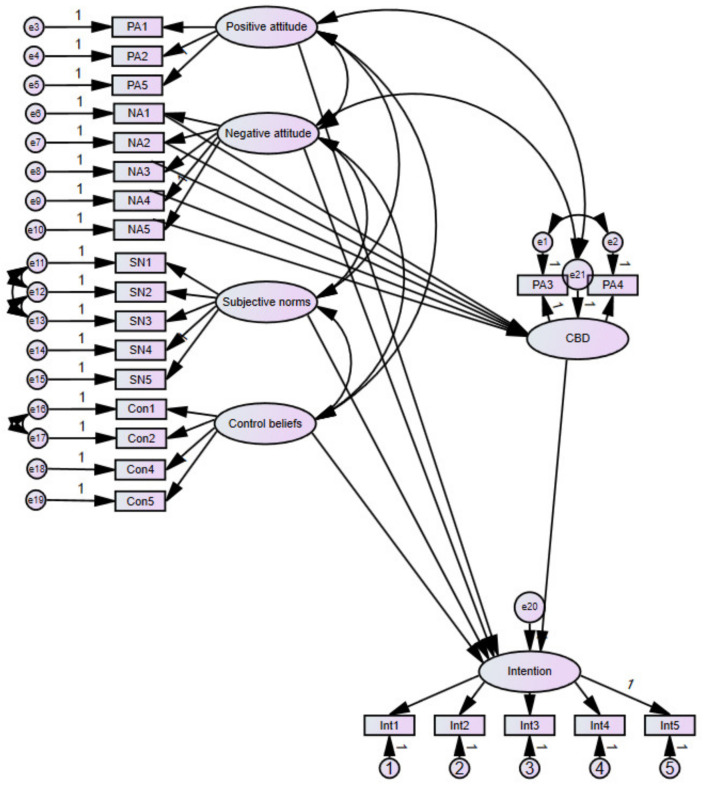
Intention to consume hemp food using the Theory of Planned Behaviour.

Structural equation modeling was performed to evaluate the influence of attitude, subjective norms, and control beliefs on intention to consume hemp food applying TPB principles (SEM sample, *n* = 766). The CBD construct was initially added to the model as a mediator of negative attitude predicting intention, however, the SEM model was reported as unidentified. Inspection of the output revealed that CBD had a positive correlation with three of the negative attitude items (NA1, NA3, NA5) and a negative correlation with the remaining two (NA2, NA4) yet all five of the negative attitude items had significant positive correlations with each of the other four items on the negative attitude scale and were found to load on a single factor during EFA. The relationships between each of the individual negative attitude items and the CBD construct required further exploration and the model was amended to represent CBD as a mediator for each of the individual items within the negative attitude construct. The model was subsequently found to be a good fit to the data; RMSEA = 0.063 (*CI* = 0.058–0.067), NFI = 0.948, TLI = 0.952, CFI = 0.960. Again the Chi-square fit statistic was significant (Chi-square = 917.295, *p* < 0.001). Although again attributed to the large sample size a further evaluation of this value for sample size (*n* = 766) and degrees of freedom (*df* = 229) as described in [23] (pp. 75–77) against a tabled chi-square fit statistic indicated an acceptable model fit at the 0.05 level. The final model is presented in Figure 2 and standardized regression weights for the model are listed in Table 6. As can be seen in the table, CBD mediated the relationship between each of the negative behavioral beliefs that formed a negative attitude, with the intention to consume hemp food.

## 4. Discussion

The use of TPB as a theoretical framework for the evaluation of acceptance of a novel food is not a new concept, however, evaluating the behavioral beliefs that form attitudes toward the food as discrete positive and negative entities is a new approach. Negative attitudes toward factors related to a novel food have been previously reported as significant to their acceptance [2,8,20]. For example, [2] included negative attitude as a discrete construct in their study of acceptance of insects as a novel food. The current study accepted empirical evidence of the importance of negative factors in the evaluation of novel foods and developed discrete positive and negative attitudinal constructs from qualitative surveys of consumer behavioral beliefs. This approach proved beneficial to understanding the acceptance of hemp food as the negative attitude items while loading onto a single factor and correlating positively with each other, had differential effects on the construct associated with the psychological benefits of Cannabidiol (CBD) oil. Originally developed as a component of positive attitudes, anomalies detected early in the analysis of the data led to the construct defined as relating to CBD being identified as a potential mediator between negative attitudes and intention to consume under the TPB framework. While the negative association of the CBD constructs with three of the items was theoretically and statistically supported, the positive association with two of the items (NA1: Eating hemp food might result in a positive roadside drug test; NA5: People don’t know enough about the effects of eating hemp food) was counterintuitive and is discussed below.

In the qualitative phase of the study, a perception of an association between hemp food and CBD oil provided an impetus for the development of the items relating to the psychological benefits of CBD oil. Reduced anxiety and increased relaxation were perceived by survey participants as a positive aspect of hemp food through this association. While these benefits are not promoted as applicable to hemp food, it is the perception of the benefits through association with CBD oil that contributes to a consumer’s intention to consume the food. What has been revealed through the application of SEM, however, is a differential influence of the perception of CBD on intention to consume hemp food, dependent upon which negative aspect of attitude is being addressed. On further examination, there appear to be three possible interpretations of the findings. The first is that despite the association of hemp food with CBD oil being perceived as a positive aspect of the product, there remains a belief that; a: There remains an association with both THC and CBD and a subsequent belief that potential for testing positive to THC after consumption of hemp food exists, and b: That not enough is known about the effects of eating hemp food. This association would be potentially problematic for the hemp food industry as it may suggest that despite assurances from industry and government sources that THC in hemp food is below detectable levels [12], consumer acceptance of this assertion appears to be low. The overall mediating effect of the association of hemp food with CBD oil, however, has a positive effect on the intention to consume hemp foods and can therefore be viewed as benefitting hemp food acceptance. While this may indicate that an association with CDB oil has thus far influenced the acceptance of hemp food, the long-term outcome from such an association would not be beneficial. It has been demonstrated that knowledge of hemp food may be lacking within the marketplace but as consumer education increases and the distinction between CBD oil and hemp food becomes more apparent, any positive influence the misperception has had to that point may be reversed.

The second interpretation is that a perception of anxiolytic and relaxation benefits in hemp food is attributed to a direct association with THC and purported side effects of illicit and “medicinal” use of marijuana. However, while this would explain the relationship between each of the negative attitudes and the CBD construct, it would only account for the increase in intention to consume hemp food if consumers were hopeful of and actively seeking THC in hemp food. While this may potentially apply to a particular subset of the population it is less likely to be discerned in a sample that has been deemed representative of the Australian population.

The third interpretation may simply be that for at least some consumers, there is confusion surrounding the differences between CBD oil, THC, and hemp oil and food which results in the anomalies in the way that the CBD related survey items were interpreted by the consumer, the perception of it as a positive or negative aspect dependent upon whether the two entities are viewed as having different or similar properties. The more likely explanation might be somewhat a combination of the first and third scenarios and suggests the challenge for the hemp food industry to separate hemp food from any association with either CBD oil or THC, whether this is through increased consumer education or the development of strains of *Cannabis sativa* which are guaranteed completely void of THC. Knowledge of the processes of varietal development is beyond the scope of this research and the authors acknowledge that the latter may not even be possible.

A positive attitude did not predict the intention to consume hemp food. That is, despite a belief in the physical and psychological benefits of hemp food, a positive attitude toward hemp food did not play a significant role in intention to consume it. The factor which provided the greatest contribution to intention to consume hemp food was consumers’ normative beliefs. Subjective norms focused on consumers’ perceptions of what family, friends, peers and doctors would think of them consuming hemp food and whether they felt others like them would consume it as well. A high level of concern for the opinion of others and the mediating effect of the CBD construct on negative attitudes suggests that for this sample of the Australian population there remains a widespread stigma associated with hemp consumption. This stigma may be difficult to eradicate as it is the artifact of the previous illegal status of hemp food combined with decades of propaganda against cannabis use in any form [14,24]. Consumers may find it difficult to disassociate hemp food from the more illicit uses of cannabis and seek the approval of others for confirmation of the acceptability of consuming hemp products. The normative beliefs and the mediated influence of negative beliefs through association with CBD oil are the only contributing factors to intention to consume hemp food in this study.

A final analysis of the findings from across the two phases of this research consisted of a meta-inference of qualitative with the quantitative findings under a pragmatic framework. Here it was revealed that the application of the theory of planned behavior and SEM in the quantitative phase two of this study was consistent with the outcomes of the initial interviews. Survey participants in the qualitative phase of the current study generally held positive attitudes toward hemp food and indicated they were likely to consume them at some time in the future, however, they also made contradictory statements indicating their reluctance to consume them at a time when they had to drive a car or attend their workplace, for example, yet denied they believed they would test positive. The issue of an association of hemp food with CBD oil and THC is complex and may not be easily resolved.

Limitations and Future Research

This research evaluates the acceptance of hemp food in the Australian population and evaluates behavioral and normative beliefs within a population where consumption of cannabis products has been illegal until recent times. The attitudes and beliefs surrounding cannabis may not extrapolate to a population where cannabis is a legal entity, or where hemp foods have been available for longer periods of time. However, the protocol of assessing negative attitudes as a separate construct under TPB may have utility for future research of alternate novel foods where highly salient negative associations are made to the food from external sources.

The findings from the current study suggest some ambiguity may exist within the population between CBD oil and THC, the two more widely known properties of cannabis, and its association with hemp food, specifically hemp seed oil. Despite being highly conspicuous of hemp food, consumers may not be fully versed in their properties, effects, health benefits, etc., or aware of the differences between *Cannabis sativa* used in hemp food production, and *Cannabis indica* which is better known as marijuana. This may have contributed to the responses regarding the anxiolytic and relaxation effects of hemp food. Future research could focus on consumer understanding of hemp food’s specific properties and improve consumer education to address the identified lack of understanding. This may be of benefit to both the hemp food industry, and the medicinal cannabis quarter.

The findings also point to a potential for the development of a strain of cannabis fit for human consumption which is free from THC. While it is beyond the scope of this research and the competency of the researchers to suggest how or if this might be achieved, it would appear that innovation in food production is currently achieving goals never before imagined. Genetic modification, 3D printed foods, and foods produced using ultrasound or infusion heat treatment, for example, are highly advanced production methods for novel foods that may only be the tip of the innovation in the food production iceberg.

## 5. Conclusions

Hemp food is unlike any other novel food insofar as it has both positive and negative associations with alternate uses for the plant from which it is derived. Despite this, however, it seems that attitudes based on these associations do not extrapolate to intention. As a result, consumers become reliant on perceptions of others’ acceptance of hemp food as a deciding factor in their intention to consume it. In the case of hemp food, its association with CBD oil has the greatest degree of complexity. It may be that large-scale acceptance of hemp food as a staple in Australia might be achieved only through the disassociation of hemp food from both CBD oil and THC. Suggested methods of achieving this are through increased consumer education or alternately the development of a strain of cannabis specifically for human consumption which is guaranteed free of any trace of THC.

The finding that highly salient associations between hemp food and other cannabis use increase the complexity of the acceptance of the food may extrapolate to other innovative food products and production methods. As innovation increases, it might become increasingly difficult to predict consumer response. Evaluating highly salient aspects as discrete entities might provide some resolution.

## Figures and Tables

**Table 1:** Internal reliability statistics for TPB items in soft launch dataset.

Construct	Cronbach’s Alpha	*n*
Positive attitude	0.867	5
Negative attitude	0.872	5
Subjective norms	0.914	5
Perceived control	0.534	5
Intention to consume	0.982	5

**Table 2:** Descriptive statistics for each of the datasets by analysis type.

	EFA	CFA	SEM
*n*	731	727	766
Male	325	294	322
Female	406	433	444
Age (*SD*) years	43.24 (15.59)	42.77 (14.73)	42.54 (14.44)
Education			
<year 12	104	84	79
Year 12 cert	158	171	170
Trade/cert IV	198	196	223
Bachelor	186	178	189
Post Grad	71	82	90
Doctorate	14	16	15

**Table 3:** Internal reliability statistics (Cronbach’s alpha) for Theory of Planned Behaviour constructs for each sample.

Construct	EFA	CFA	SEM	*n*
	α	α	α	
Positive attitude	0.847	0.854	0.861	5
Negative attitude	0.858	0.877	0.874	5
Subjective norms	0.893	0.904	0.883	5
Perceived control	0.520	0.595	0.607	4
Intention to consume	0.977	0.980	0.979	5

EFA = Exploratory factor analysis *n* = 731; CFA = Confirmatory factor analysis *n* = 727; SEM = Structural equation modelling *n* = 766.

**Table 4:** Theory of Planned Behaviour response items by construct represented.

Construct	Item	Statement
Positive attitude	PA1	Eating hemp food is healthy
	PA2	Eating hemp food is good for my diet
	PA3	Eating hemp food would make a person feel more relaxed
	PA4	Eating hemp food would likely reduce anxiety
	PA5	There are many benefits to eating hemp food
Negative attitude	NA1	Eating hemp food might result in a positive roadside drug test
	NA2	Eating hemp food might have some negative physical effects
	NA3	Eating hemp food might reduce a person’s concentration at work
	NA4	Eating hemp food might negatively impact a person’s mental health
	NA5	People don’t know enough about the effects of eating hemp food
Subjective norms	SN1	My family would think it is okay for me to consume hemp food
	SN2	My friends would think it is okay for me to consume hemp food
	SN3	My peers would approve if I consumed hemp food
	SN4	Many people that are similar to me would be likely to consume hemp food
	SN5	Doctors would be pleased if more people consumed hemp food
Perceived control	Con1	I am free to choose the food I wish to eat
	Con2	Choosing to eat hemp food is a choice I am free to make
	Con3	I have seen a place where hemp food is sold ◆
	Con4	I am able to afford the food choices I make
	Con5	My own health conditions would not overly restrict my choice to consume hemp food
Intention to consume	Int1	I am likely to eat hemp food
	Int2	I intend to eat hemp food
	Int3	I would like to introduce hemp food into my diet
	Int4	I want to eat hemp food
	Int5	I plan to eat hemp food in the near future

◆ Item Con3 was removed before final analysis.

**Table 5:** Covariance and correlation of CBD construct and positive and negative attitude.

		Covariance	Correlation
CBD	Positive attitude	0.933 ***	0.489
CBD	Negative attitude	0.131 ***	0.165
Positive attitude	Negative attitude	−0.399 ***	−0.498

*** *p* < 0.001.

**Table 6:** Standardized regression weights for intention to consume hemp food.

			Estimate
NA5	<	Negative attitude	0.366
NA4	<	Negative attitude	0.915
NA3	<	Negative attitude	0.966
NA2	<	Negative attitude	0.872
NA1	<	Negative attitude	0.726
CBD	<	NA1	0.452
CBD	<	NA2	−0.176
CBD	<	NA3	−0.883
CBD	<	NA4	−0.566
CBD	<	NA5	0.082
Intention	<	CBD	0.123
Intention	<	Control beliefs	−0.018
Intention	<	Subjective norms	0.921
Intention	<	Negative attitude	−0.061
Intention	<	Positive attitude	−0.108
PA5	<	Positive attitude	0.889
PA2	<	Positive attitude	0.920
PA1	<	Positive attitude	0.885
SN5	<	Subjective norms	0.664
SN4	<	Subjective norms	0.807
SN3	<	Subjective norms	0.756
SN2	<	Subjective norms	0.760
SN1	<	Subjective norms	0.743
Con5	<	Control beliefs	0.748
Con4	<	Control beliefs	0.316
Con2	<	Control beliefs	0.579
Con1	<	Control beliefs	0.279
PA3	<	CBD	0.610
PA4	<	CBD	0.673
Int5	<	Intention	0.948
Int4	<	Intention	0.956
Int3	<	Intention	0.947
Int2	<	Intention	0.972
Int1	<	Intention	0.925

## Data Availability

The data presented in this study are available on request from the corresponding author. The data are not publicly available as it forms a substantial component of a yet-to-be-submitted doctoral thesis from which additional papers are expected to be compiled.
